# Metabolic Dysfunction-Associated Steatotic Liver Disease in People Living With HIV/AIDS: Unraveling the Paradox of Lean Phenotype and Fibrosis

**DOI:** 10.7759/cureus.85415

**Published:** 2025-06-05

**Authors:** Antony George, Prasanth Thayyil Sudheendran, Jijo Varghese, Shanid A, Jithin John, Nuzil Moopan, Jacob Raja AS, Aditya Verma, Srijaya Sreesh, Krishnadas Devadas

**Affiliations:** 1 Gastroenterology and Hepatology, Government Medical College, Thiruvananthapuram, IND; 2 Gastroenterology and Hepatology, NS Co-operative Hospital, Kollam, IND

**Keywords:** lean masld, masld, plhiv, primary masld, significant liver fibrosis

## Abstract

Background and aim: Metabolic dysfunction-associated steatotic liver disease (MASLD) has emerged as the leading cause of liver-related morbidity and mortality in people living with HIV/AIDS infection (PLHIV), with the advent of newer antiretroviral drugs. However, the metabolic alterations, biochemical analysis, and associated significant fibrosis, when compared to primary MASLD, remain poorly understood. We conducted a study comparing PLHIV with MASLD and primary MASLD subjects.

Methods: We enrolled 120 consecutive PLHIV with MASLD and 120 age- and sex-matched primary MASLD patients based on the MASLD diagnostic criteria. All patients underwent evaluation of metabolic parameters, anthropometric assessment, ultrasonography, and transient elastography. Significant and advanced fibrosis was defined as transient elastography (TE)≥ 8.2 kPa and ≥ 9.7 kPa, respectively.

Results: The metabolic risk factors (diabetes mellitus, systemic hypertension, dyslipidemia, and metabolic syndrome) were comparable between the two groups. "Lean" MASLD was more prevalent in the PLHIV group (p<0.001). Hand-grip strength was comparable among groups (p=0.728). Visceral fat (p<0.001), BMI (p<0.001), waist circumference (p<0.001), hip circumference (p<0.001), and waist-hip ratio (p=0.004) were significantly higher in the primary MASLD group. Significant and advanced fibrosis was higher in the PLHIV with MASLD compared to the primary MASLD group (p=0.026).

Conclusion: Despite having a comparable metabolic profile and lower body fat, PLHIV tends to have conspicuously higher "lean" MASLD and more significant or advanced liver fibrosis compared to primary MASLD. There is an unmet need for active screening and aggressive treatment of PLHIV with MASLD, mainly because they tend to have a higher number of "lean" MASLD.

## Introduction

Human immunodeficiency virus (HIV) infection poses a major public health problem around the globe. The latest estimates report a prevalence of 39.9 million (36.1 million-44.6 million) people living with HIV/AIDS (PLHIV) worldwide in 2023 [1], whereas, in India, the estimated prevalence was 23.48 lakhs (17.98 lakhs - 30.98 lakhs) in 2019 [2]. Since the advent of highly active antiretroviral treatment (HAART) and its widespread availability, the prognosis for HIV-infected patients has improved dramatically. Even though AIDS-related mortality has reduced, liver disease-related morbidity and mortality are becoming an increasingly important issue in PLHIV [3,4]. Now, liver disease is the most common non-AIDS-related cause of death among HIV-infected patients [5]. Historically, viral hepatitis and alcohol intake were the major contributors to liver-related mortality in PLHIV. With the advent of antiretroviral drugs, the burden of liver disease due to viral hepatitis has steadily been on the decline. In this context, metabolic dysfunction-associated steatotic liver disease (MASLD) is a growing concern due to its morbidity and mortality.

Various studies have reported a higher prevalence of MASLD in PLHIV compared to the general population [4,6]. The majority of the risk factors for the development of MASLD in PLHIV are similar to the ones implicated in the development of MASLD in the general population, like diabetes mellitus, systemic hypertension, dyslipidemia, and obesity, all being components of the metabolic syndrome [7-9]. The risk of metabolic syndrome in patients with HIV is nearly twice that in the general population, with a reported prevalence of 15-60% [10]. HIV-related risk factors can also contribute to the development of MASLD. Nucleotide reverse transcriptase inhibitors, especially Didanosine and Stavudine, have been implicated in the development of MASLD due to their effect on lipid metabolism via mitochondrial toxicity [11]. Recent studies have shown evidence of increased gut permeability and, thereby, increased bacterial translocation in chronic HIV patients, which can lead to hepatic inflammation [4,12]. HAART-associated lipodystrophy, which is associated with zidovudine, stavudine, and didanosine among others, develops in conjunction with insulin resistance and dyslipidemia and plays a role in the development of MASLD in PLHIV [13]. HIV infection, per se, has been incriminated in the development of fatty liver. The proposed mechanisms include mitochondrial dysfunction, hypertriglyceridemia, lower low-density lipoprotein (LDL), and higher cholesterol [14,15].

The importance of MASLD, in any given population, is the progression of the disease from simple fatty liver to MASH, MASH-cirrhosis, and hepatocellular carcinoma. The transition from MASLD to MASH is considerably higher in patients with HIV infection, compared to the general population, with multiple factors contributing to it. Chronic inflammation and excessive cytokine production, which are hallmarks of HIV infection, could contribute to the progression of MASLD [16]. A recent meta-analysis reported a 35% prevalence for MASLD in PLHIV [17]. Moreover, patients with HIV infection and MASLD have also been reported to have a higher prevalence of fibrosis. Very few studies have been conducted in India in this regard, despite the high number of HIV patients.

Despite the high prevalence of MASLD in the HIV-infected population, little is known about the clinical, biochemical, and histopathological traits of HIV-associated MASLD and whether or not it differs from MASLD in the general population or primary MASLD, in terms of clinical presentation and severity. Our aim was to study the differences between primary MASLD and HIV-associated MASLD in terms of various metabolic, biochemical, anthropometric, and non-invasive liver fibrosis markers. This article was previously presented as an abstract/poster at the 31st Annual Meeting of the Indian National Association for the Study of the Liver (INASL) held on August 4, 2023, in Bhubaneswar, India. A sub-study of the article was presented as an oral paper at the Asia Pacific Digestive Week 2023 held in Bangkok, Thailand, from December 6, 2023, to December 9, 2023.

## Materials and methods

One hundred and twenty consecutive PLHIV with MASLD attending the anti-retroviral treatment (ART) center and 120 age- and sex-matched HIV-negative patients with MASLD, attending the MASLD Clinic, Government Medical College Hospital, Thiruvananthapuram, Kerala, India, were prospectively enrolled in this cross-sectional observational study over a one-year period. The study commenced on April 1, 2022, and was completed by March 2023. All patients were diagnosed with MASLD by ultrasonography using the MASLD diagnostic criteria [18] and after ruling out secondary causes of steatosis. We excluded patients with significant alcohol consumption (> 20g/day for both men and women), positive serum markers of Hepatitis B or C infection, positive simplified Autoimmune Hepatitis score, and history of drug intake which are likely to cause secondary steatosis, low ceruloplasmin and increased 24-hour urine copper and high serum ferritin and transferrin saturation. Clearance was obtained from the National AIDS Control Organisation (1822/M&E/2020/KSACS) and the Human Ethics Committee, Medical College, Thiruvananthapuram (HEC NO: 04/29/2022/MCT; dated May 31, 2022). A qualified gastroenterologist counseled all patients regarding their involvement in the study. Informed consent was taken in the vernacular.

Demographic and clinical data

Detailed history and physical examination, as well as anthropometry and standard blood investigations, were assessed in both groups. Fibrosis-4 (FIB-4), AST to Platelet Ratio Index (APRI), and NAFLD Fibrosis Score (NFS) were calculated. Visceral fat was measured using the bioelectric impedance analyzer (Dr Trust USA Smart Body Fat and Body Composition Rechargeable Scale Analyser 2.0, Dr Trust, New York, USA).

Diagnosis of hepatic steatosis

Supersonic Imagine Aixplorer Ultimate ultrasound machine (SuperSonic Imagine S.A., Aix-en-Provence, France) with a 3.5 MHz convex transducer was used for imaging purposes to diagnose fatty liver. The echogenicity of the right kidney was used to compare and determine the liver parenchymal echogenicity. Grading of hepatic steatosis was based on the contrast between the liver and the kidney, the sonographic appearance of the intrahepatic vessels, liver parenchyma, and the diaphragm.

Assessment of fibrosis

Fibrosis assessment was done using the Fibroscan device (Echosens, Paris, France), which uses a 50-MHz wave passed into the liver from a small transducer. Evaluations with at least 10 valid measurements, a kPa inter-quartile range <30% of the median, and a successful acquisition rate of ≥60% were included in the analysis. Based on the expert panel recommendations by Younossi et al. in 2021, significant fibrosis and advanced fibrosis were defined as vibration-controlled transient elastography (VCTE) values ≥8.2 kPa and ≥9.7 kPa, respectively [19]. 2D shear wave elastography (SWE) was also assessed using the linear probe of the Supersonic Imagine Aixplorer Ultimate ultrasound machine (SuperSonic Imagine S.A., France). Validated non-patented scoring systems were calculated, including APRI, FIB-4, and NFS scores.

Statistical analysis

Continuous data were expressed as means, standard deviations, or medians with interquartile ranges based on the tests of normality. The normality of data was tested using the Kolmogorov-Smirnov test. Categorical data were expressed as frequencies and percentages. We compared the various metabolic, anthropometric, biochemical, and non-invasive fibrosis scores between the HIV-positive and HIV-negative MASLD groups. Continuous variables were compared between the two groups using the Independent t-test or the Mann-Whitney U test. Categorical variables were compared between the groups using the Chi-square or Fisher's Exact Test. A p-value <0.05 was considered statistically significant. Statistical analysis was done using IBM SPSS Statistics for Windows, Version 27 (IBM Inc., Armonk, NY, USA).

## Results

One hundred and twenty consecutive PLHIV with MASLD and an equal number of age- and sex-matched primary MASLD patients were enrolled. The mean age of patients with primary MASLD was 48.2 years, whereas that of HIV patients with MASLD was 48.5 years. Of the 120 primary MASLD patients, 72 (60%) were male individuals and 48 (40%) were female individuals. There were 71 (59.2%) male patients and 49 (40.8%) female patients in the PLHIV with MASLD group. Both groups were adequately age- and sex-matched (Table 1). All PLHIV patients were on tenofovir, lamivudine, and dolutegravir (TLD regime) at the time of enrolment.

**Table 1:** Demographic and clinical characteristics between HIV-positive and HIV-negative groups MASLD: metabolic dysfunction-associated steatotic liver disease; PLHIV: people living with HIV/AIDS infection; SD: standard deviation

Variables	Primary MASLD	PLHIV + MASLD
Age (Mean ± SD)	48.2 ± 9.2 years	48.5 ± 9.4 years
Males, n (%)	72 (60)	71 (59.2)
Females, n (%)	48 (40)	49 (40.8)
Diabetes, n (%)	38 (31.7)	30 (25)
Systemic hypertension, n (%)	20 (16.7)	20 (16.7)
Dyslipidemia, n (%)	30 (25)	23 (19.2)

Comparison of metabolic alterations

Among HIV patients with fatty liver, 30 (25%) had diabetes mellitus (DM), 20 (16.7%) had systemic hypertension, and 23 (19.1%) patients had dyslipidemia. Thirty-eight (31.6%) patients had DM, 20 (16.7%) patients had systemic hypertension, and 30 (25%) patients had dyslipidemia in the primary MASLD group. The groups were similar in terms of the presence of DM (p=0.158), systemic hypertension (p=0.569), and dyslipidemia (p=0.175) (Tables 2-4).

**Table 2:** Diabetes mellitus in HIV-positive and HIV-negative groups P-values were calculated using the Chi-square test

HIV infection	Diabetes mellitus		Total	p value
	No n (%)	Yes n (%)		
No	82 (68.3)	38 (31.7)	120	0.158
Yes	90 (75)	30 (25)	120	
Total	172	68	240	

**Table 3:** Systemic hypertension in HIV-positive and HIV-negative groups P-values were calculated using the Chi-square test

HIV infection	Systemic hypertension		Total	p value
	No n (%)	Yes n (%)		
No	100 (83.3)	20 (16.7)	120	0.569
Yes	100 (83.3)	20 (16.7)	120	
Total	200	40	240	

**Table 4:** Dyslipidemia in HIV-positive and HIV-negative groups P-values were calculated using the Chi-square test

HIV infection	Dyslipidemia		Total	p value
	No n (%)	Yes n (%)		
No	90 (75)	30 (25)	120	0.175
Yes	97 (80.8)	23 (19.2)	120	
Total	187	53	240	

"Lean" MASLD was defined based on the guidelines of the Indian National Association for the Study of the Liver (INASL) [20]. "Lean" MASLD was significantly higher in the HIV with MASLD group (22.5% vs 6.7%) (p<0.001) (Table 5).

**Table 5:** Lean MASLD in HIV-positive and HIV-negative groups MASLD: metabolic dysfunction-associated steatotic liver disease P-values were calculated using the independent t-test

HIV infection	Lean MASLD		Total	p value
	No n (%)	Yes n (%)		
No	112 (93.3)	8 (6.7)	120	<0.001
Yes	93 (77.5)	27 (22.5)	120	
Total	205	35	240	

We observed that 43 (35.8%) patients with primary MASLD and 35 (29.1%) HIV patients with MASLD had features suggestive of metabolic syndrome (MetS), which was not statistically significant (p=0.167) (Table 6).

**Table 6:** Metabolic syndrome in HIV-positive and HIV-negative groups P-values were calculated using the independent t-test

HIV infection	Metabolic syndrome		Total	p value
	No n (%)	Yes n (%)		
No	77 (64.2)	43 (35.8)	120	0.167
Yes	85 (70.8)	35 (29.2)	120	
Total	162	78	240	

Comparison of anthropometric parameters

In our cohort, weight (p=0.001), Body Mass Index (p<0.001), waist circumference (p<0.001), hip circumference (p<0.001), waist-hip ratio (p=0.004) and visceral fat (p<0.001) were higher in the primary MASLD group. Height (p=0.21) and average Handgrip strength (p=0.728) were comparable between the two groups. We observed higher visceral fat levels among patients with primary MASLD when compared to secondary MASLD, which was statistically significant (p<0.001) (Table 7).

**Table 7:** Comparison of anthropometric parameters between HIV-positive and HIV-negative groups TSFT: triceps skinfold thickness; MAUC: mid-upper arm circumference; BMI: Body Mass Index P-values were calculated using the independent t-test

Anthropometric parameters	HIV infection	N	Mean	Std. deviation	p value
TSFT (mm)	No	120	17.542	6.3031	<0.001
	Yes	120	11.167	3.7154	
MAUC (cm)	No	120	29.558	3.5805	<0.001
	Yes	120	24.650	3.1050	
Hip circumference (cm)	No	120	99.733	10.9964	<0.001
	Yes	120	93.167	6.0228	
Waist circumference (cm)	No	120	97.150	11.4199	<0.001
	Yes	120	88.925	6.3208	
BMI (kg/m^2^)	No	120	27.92	4.76	<0.001
	Yes	120	23.93	2.48	
Visceral fat (%)	No	120	12.405	4.9649	<0.001
	Yes	120	9.778	2.5237	

Comparison of blood parameters

Higher aspartate aminotransferase (AST) levels were observed in HIV patients with MASLD (p=0.011). Higher serum cholesterol levels (p=0.011) and serum triglyceride levels (p<0.001) were found in the primary MASLD group. There was no difference between the groups in terms of hemoglobin (p=0.497), total count (p=0.23), platelet count (p=0.112), total and direct bilirubin (p=0.489; p=0.614), alanine aminotransferase (ALT) (p=0.101), high-density lipoprotein (HDL) (p=0.249) and low-density lipoprotein (LDL) (p=0.159) (Table 8).

**Table 8:** Comparison of blood parameters between HIV-positive and HIV-negative groups SGOT: serum glutamic-oxaloacetic transaminase; SGPT: serum glutamic pyruvic transaminase; HDL: high-density lipoprotein P-values were calculated using the independent t-test

Biochemical parameters	HIV infection	N	Mean	Std. deviation	p value
Bilirubin (total) (mg/dL)	No	120	0.7987	0.27060	0.489
	Yes	120	0.8267	0.35069	
SGOT (U/L)	No	120	37.075	15.6136	0.011
	Yes	120	46.033	34.6776	
SGPT (U/L)	No	120	52.117	30.8513	0.101
	Yes	120	61.108	51.2195	
Cholesterol (mg/dL)	No	120	218.458	27.1129	0.011
	Yes	120	209.508	26.6682	
Triglyceride (mg/dL)	No	120	139.067	38.7844	<0.001
	Yes	120	120.483	31.7884	
HDL (mg/dL)	No	120	43.925	7.3965	0.249
	Yes	120	42.558	10.6256	

Comparison of fibrosis

Thirty (25.8%) patients in the PLHIV with MASLD group had significant fibrosis compared to 23 (19.2%) patients in the primary MASLD group (p=0.026). Comparing fibrosis parameters between the two groups, we observed that HIV patients with MASLD had higher VCTE (p=0.029), 2D-SWE (p<0.001), APRI score (p=0.035), and FIB-4 score (p=0.015). But, primary MASLD patients had higher NFS scores (p<0.001) (Table 9).

**Table 9:** Comparison of fibrosis between the HIV-positive and HIV-negative groups TE: transient elastography; SWE: shear wave elastography; NFS: Non-Alcoholic Fatty Liver Disease Fibrosis Score; FIB4: Fibrosis-4; APRI: AST to Platelet Ratio Index; kPa: kilopascal P-values were calculated using the independent t-test

Liver fibrosis markers	HIV infection	N	Mean	Std. deviation	p value
TE (kPa)	No	120	7.394	1.6114	0.026
	Yes	120	7.854	1.5767	
SWE (kPa)	No	120	6.796	1.4071	<0.001
	Yes	120	7.540	1.4246	
NFS	No	120	-2.293	1.209	<0.001
	Yes	120	-2.849	1.2152	
FIB4	No	120	0.97027	0.39911	0.017
	Yes	120	1.1585	0.76309	
APRI	No	120	0.36153	0.19348	0.035
	Yes	120	0.47625	0.56025	

## Discussion

We observed that metabolic alterations like DM, systemic hypertension, and dyslipidemia were comparable between the two groups. Metabolic syndrome (MetS) was observed more in the primary MASLD group, but there was no significant difference between the two groups. A few studies have also shown a significant association between MASLD in HIV patients and DM [21,22].

Primary MASLD is widely considered to be a hepatic manifestation of MetS [23]. Though traditionally, HIV patients have been known to have lower BMI when compared to primary MASLD, the presence of MetS is associated with MASLD [21,24]. We observed no significant differences in the metabolic factors, including metabolic syndrome, between the two groups. This could be due to the high prevalence of obesity and metabolic syndrome in our region, as seen in the study by Chalmers et al. [25].

"Lean" MASLD represents an outlier in the phenotypic spectrum of MASLD. INASL 2023 guidelines defined "lean" MASLD as patients with hepatic steatosis and BMI <23 kg/m2 with waist circumference <90 cm in men and <80 cm in women [20]. We found that "lean" MASLD was significantly higher in PLHIV compared to primary MASLD as reported by De et al. [26]. It has also been reported that "lean" MASLD is not a benign condition as previously thought, with a higher prevalence of MASH [27]. Moreover, most of the existing therapeutic options target obese MASH. Clinical trials of "lean" MASH are lacking. HIV patients with "lean" MASLD should be actively worked up for the presence of MASH, and aggressive treatment should be initiated. Therefore, there is an unmet need for the diagnosis and management of "lean" MASH in HIV patients.

Assessment of anthropometry showed that the primary MASLD group had significantly higher weight, BMI, waist circumference, hip circumference, waist-hip ratio, MUAC, TSFT, and visceral fat. This suggests that the primary MASLD group has higher subcutaneous and visceral fat when compared to PLHIV with MASLD. The handgrip strength was comparable between the two groups, suggesting that muscle strength was nearly equal in the study population.

The PLHIV with the MASLD group had significantly higher SGOT levels compared to the primary MASLD group, whereas no significant difference was observed in SGPT levels. This could be due to the higher prevalence of significant fibrosis levels in the PLHIV group. Ahmed et al. observed that higher SGOT levels were seen in higher stages of fibrosis, whereas SGPT levels did not correlate with fibrosis [28]. We also observed that the PLHIV group had significantly lower cholesterol and lower triglyceride levels, echoing the findings reported by Ghadir et al. in patients with advanced fibrosis [29].

Among PLHIV with MASLD, 25.8% had significant liver fibrosis compared to 19.2% in the primary MASLD group, which was statistically significant. On analyzing other non-invasive parameters of liver fibrosis assessment, we observed that SWE, APRI, and FIB-4 were significantly higher in the PLHIV group, whereas NFS (p<0.001) was lower in the PLHIV group. The NFS score incorporates BMI, which was lower in the PLHIV group.

Limitations

Our study was conducted at a single center, with subjects from the same ethnicity. Another limitation is the relatively small sample size, which may limit the generalizability of the findings. Therefore, the results may not be extrapolated to the entire population. A multi-center study could help alleviate this limitation. Also, liver biopsy, which could have added more valuable information, was not available in our study population. The CD4 count was not available for our patients, which could have enhanced the significance of the findings.

## Conclusions

With the advent of newer antiretroviral treatment regimes, the longevity of PLHIV has dramatically changed over the past decade. MASLD has emerged as the primary cause of liver-related morbidity and mortality in PLHIV. Our age- and sex-matched study showed that despite having a comparable metabolic profile, PLHIV patients with MASLD tend to have conspicuously higher "lean" body type and significant fibrosis compared to primary MASLD.

Thus, there is an unmet need for active screening and aggressive treatment of PLHIV with MASLD, especially "lean" MASLD. Since the incidence of "lean" MASLD and advanced fibrosis is higher in PLHIV patients, and "lean" MASLD is not amenable to dietary modification and exercise, studies focusing on pharmacotherapy for "lean" MASLD in PLHIV need to be done. Addressing this gap could significantly improve long-term hepatic outcomes in PLHIV.
